# Trends in primary care blood tests prior to lung and colorectal cancer diagnosis—A retrospective cohort study using linked Australian data

**DOI:** 10.1002/cam4.70006

**Published:** 2024-07-13

**Authors:** Meena Rafiq, Allison Drosdowsky, Ben Solomon, Marliese Alexander, Peter Gibbs, Gavin Wright, Justin M. Yeung, Georgios Lyratzopoulos, Jon Emery

**Affiliations:** ^1^ Department of General Practice and Centre for Cancer Research University of Melbourne Melbourne Victoria Australia; ^2^ Epidemiology of Cancer Healthcare & Outcomes (ECHO) Group, Department of Behavioural Science and Health Institute of Epidemiology and Health Care (IECH), UCL London UK; ^3^ Peter MacCallum Cancer Centre Melbourne Victoria Australia; ^4^ Walter and Eliza Hall Institute of Medical Research Parkville Victoria Australia; ^5^ Department of Surgery, Western Precinct University of Melbourne Melbourne Victoria Australia

**Keywords:** blood tests, colorectal cancer, early diagnosis, general practice, lung cancer

## Abstract

**Introduction:**

Abnormal results in common blood tests may occur several months before lung cancer (LC) and colorectal cancer (CRC) diagnosis. Identifying early blood markers of cancer and distinct blood test signatures could support earlier diagnosis in general practice.

**Methods:**

Using linked Australian primary care and hospital cancer registry data, we conducted a cohort study of 855 LC and 399 CRC patients diagnosed between 2001 and 2021. Requests and results from general practice blood tests (six acute phase reactants [APR] and six red blood cell indices [RBCI]) were examined in the 2 years before cancer diagnosis. Poisson regression models were used to estimate monthly incidence rates and examine pre‐diagnostic trends in blood test use and abnormal results prior to cancer diagnosis, comparing patterns in LC and CRC patients.

**Results:**

General practice blood test requests increase from 7 months before CRC and 6 months before LC diagnosis. Abnormalities in many APR and RBCI tests increase several months before cancer diagnosis, often occur prior to or in the absence of anaemia (in 51% of CRC and 81% of LC patients with abnormalities), and are different in LC and CRC patients.

**Conclusions:**

This study demonstrates an increase in diagnostic activity in Australian general practice several months before LC and CRC diagnosis, indicating potential opportunities for earlier diagnosis. It identifies blood test abnormalities and distinct signatures that are early markers of LC and CRC. If combined with other pre‐diagnostic information, these blood tests have potential to support GPs in prioritising patients for cancer investigation of different sites to expedite diagnosis.

## Graphical abstract

This study shows there is an increase in diagnostic activity in Australian general practice several months before lung and colorectal cancer diagnosis, indicating potential opportunities for earlier diagnosis. It identifies blood test abnormalities and distinct signatures that are early markers of these cancers. If combined with other pre‐diagnostic information, these blood tests have potential to support GPs in prioritising patients for cancer investigation of different sites to expedite diagnosis.

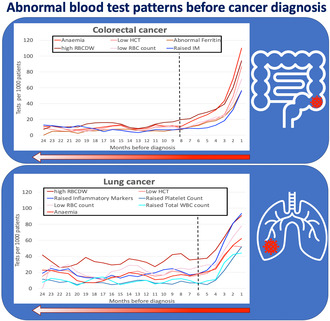

## 1 INTRODUCTION

Lung cancer (LC) and colorectal cancer (CRC) are two of the five most common cancers in the world. In Australia, they account for 9% and 10% of all new cancer diagnoses each year respectively.^1^ Despite advances over recent decades, around 50% of LC and 40% of CRC patients in Australia are still diagnosed at a late stage,^2^ which is associated with lower survival.^3^, ^4^, ^5^ Consequently, LC and CRC remain the two leading causes of cancer death in Australia.^1^ Diagnosing LC and CRC earlier is essential to produce a stage shift and improve patient outcomes and experience. As most cancer patients are diagnosed after presenting with symptoms to primary care,^6^ even where screening programs exist, research is needed to determine if there are opportunities for earlier LC and CRC diagnosis in general practice.

Previous UK and Danish studies have shown there is increased diagnostic activity in general practice in the year preceding LC and CRC diagnosis. This indicates potential opportunities exist to expedite cancer diagnosis if these patients can be identified. In these studies, general practitioner (GP) consultation rates increased 9 months prior to CRC diagnosis,^7^ with associated pre‐diagnostic increases in haemoglobin test requests and prescriptions for anti‐haemorrhoidal medications, laxatives and proton pump inhibitors.^7^, ^8^, ^9^ GP consultations increased 4 months prior to LC diagnosis,^10^ accompanied by increases in x‐ray requests, lung function tests, and prescriptions for antibiotics, inhalers and cough suppressants.^9^, ^10^ A small number of common blood test abnormalities have been found to increase in the lead up to LC and CRC diagnosis. These include increased rates of anaemia, raised inflammatory markers and thrombocytosis up to 9 months before CRC diagnosis^11^ and increases in mean CRP and platelet levels in the year preceding LC diagnosis.^12^ These abnormalities may be predictors of as‐yet‐undetected cancer^13^, ^14^, ^15^, ^16^ and could help to diagnose patients earlier, particularly in the diagnostically challenging group of around half of LC and CRC patients who present with non‐specific symptoms.^17^, ^18^

It is unknown if similar increases in primary care diagnostic activity and blood test abnormalities occur before LC and CRC diagnosis in the other healthcare settings, such as Australia, where the primary healthcare system, access to diagnostic tests, population demographics and GP/patient help‐seeking behaviours differ from Denmark and England. Knowledge is also needed on whether any additional blood test abnormalities occur pre‐diagnosis that could be deployed for earlier identification of LC and CRC patients. This is needed to support earlier diagnosis, as blood test abnormalities are common and in isolation their predictive value for detecting cancer will be low.

We used linked Australian primary care and hospital cancer registry data to examine trends in GP blood test requests and results over time prior to LC and CRC diagnosis. We aimed to identify when GP healthcare use first starts to increase pre‐diagnosis and what blood test abnormalities could be early markers of LC and CRC. Furthermore, we aimed to investigate whether pre‐diagnostic patterns of blood test abnormalities differed among patients with LC and CRC.

## 2 MATERIALS AND METHODS

### 2.1 Design and datasets

**FIGURE 1:**
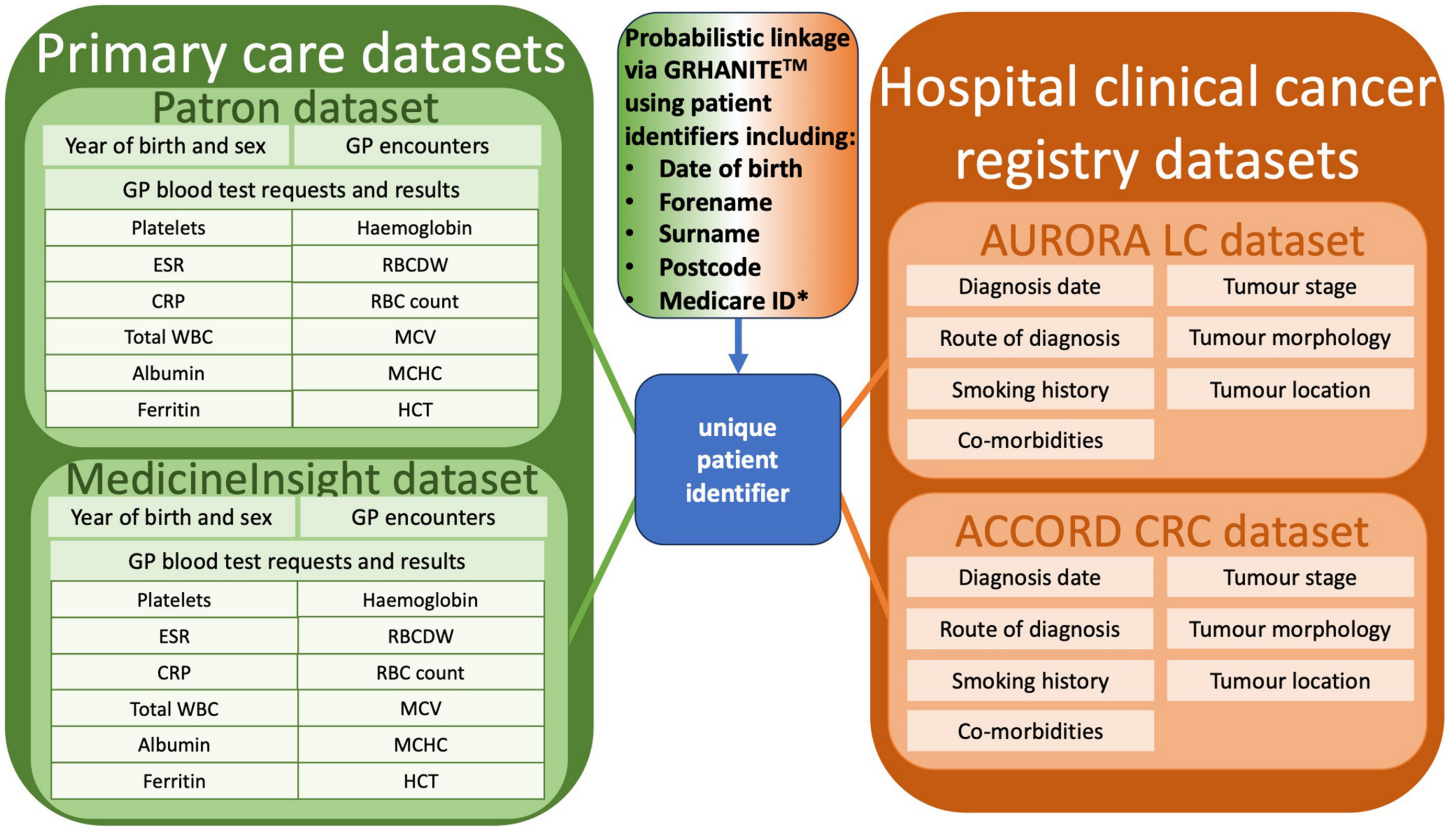
Linked data sources used in the study and variables extracted from each source. *5 digits of Medicare ID; ACCORD, Australian Comprehensive Cancer Outcomes and Research Database; AURORA, AUstralian Registry and biObank of thoRAcic cancers; CRC, colorectal cancer; CRP, c‐reactive protein; ESR, erythrocyte sedimentation rate; GP, general practice; HCT, haematocrit; LC, lung cancer; MCHC, mean cell haemoglobin concentration; MCV, mean cell volume; RBC, red blood cell; RBCDW, red blood cell distribution width; WBC, white blood cell count.

We conducted a longitudinal cohort study using Australian linked primary care and hospital cancer registry data^19^ obtained via BioGrid,^20^ a research service that specialises in linking datasets. Patients with a new diagnosis of LC or CRC were identified from two hospital based clinical cancer registries: for LC, the AUstralian Registry and biObank of thoRAcic cancers (AURORA) using the Peter MacCallum Cancer Centre and St Vincent's Hospital datasets; and for CRC, the Australian Comprehensive Cancer Outcomes and Research Database (ACCORD) using the Royal Melbourne Hospital and Western Health datasets^21^ (Figure 1). AURORA and ACCORD contain clinician‐recorded information relating to the diagnosis, treatment and outcomes of cancer patients.

Linked primary care data for this study were obtained from the MedicineInsight^22^, ^23^ and Patron databases^24^, ^25^ (Figure 1). MedicineInsight is a representative, nationwide Australian database of de‐identified primary care electronic health records (EHR) covering approximately 8% of all general practices in Australia.^22^ For this study a subset of the MedicineInsight dataset comprising general practices located in the state of Victoria was used. The Patron primary care dataset^24^ is operated by the Department of General Practice at the University of Melbourne and contains de‐identified patient clinical and administrative data from over 130 GP clinics in Victoria.^26^

### 2.2 Defining the outcome and study population

Individuals aged ≥18 years with a new diagnosis of LC or CRC recorded in AURORA or ACCORD between 2001 and 2021 were identified. The earliest date of cancer diagnosis recorded in the clinical registry was determined (defined in ACCORD as the date that disease was first diagnosed by histology or imaging and defined in AURORA as the date of histological diagnosis or if not available then date of clinical diagnosis). LC and CRC patients were included in the study if they had linkage to either the MedicineInsight or Patron primary care databases and had at least one GP encounter in a linked general practice in the year before cancer diagnosis. For each patient in the final study population, clinician recorded hospital registry data were extracted on date of diagnosis, tumour characteristics (stage, morphology, site), route of diagnosis (symptomatic, screening, incidental), smoking history and relevant co‐morbidities. Gold‐standard National Bowel Cancer Screening data were not available, therefore ‘screen detected’ status was determined from ACCORD, based on review of the hospital medical notes. As this group could include some symptomatic patients or patients with abnormal GP blood test results detected before they underwent screening these patients were included in the CRC cohort. Primary care data were extracted on patient demographics (age and sex), GP encounters (date) and GP blood test use and findings (date, test name, result) pre‐diagnosis.

### 2.3 Defining the explanatory variables

The following blood tests were selected a priori for inclusion in this study based on clinical knowledge and published literature^14^: Six acute phase reactant (APR) tests (platelet count, inflammatory markers [CRP and ESR—considered together], white blood cell [WBC] count, albumin and ferritin) and six red blood cell indices (RBCI) (haemoglobin, haematocrit, red blood cell [RBC] count, red blood cell distribution width [RBCDW], mean cell volume [MCV] and mean cell haemoglobin concentration [MCHC]). If patients had seen their GP at least once a year for two or more consecutive years pre‐diagnosis, data on all such tests requested in primary care were extracted for 24 months pre‐diagnosis from MedicineInsight and Patron. Otherwise, data were extracted from the first GP encounter in this period until cancer diagnosis. Data were extracted on test date, test type and test result (classified as normal or abnormal based on standard laboratory reference ranges in males and females). As raised ferritin is a marker of an acute phase response and low ferritin is a marker of iron deficiency anaemia, both raised and low ferritin were included in the analyses. Where units of measurement varied between testing laboratories, the most frequently used unit was selected and, where appropriate, results were converted (e.g. haemoglobin results in g/L were converted to g/dL by dividing by 10). Any biologically implausible results were excluded. In the event a patient had more than one test result for the same test on the same day, only one test was included to prevent duplicate counting, and the mean value of the results was used.

### 2.4 Statistical analysis

The baseline characteristics of the study population were examined, with sensitivity analyses conducted comparing patient characteristics with (a) LC and CRC patients in the clinical registries without linkage to a primary care dataset and (b) State‐wide data from the Victorian Cancer Registry on LC and CRC patients from 2016, to ensure they were comparable.^27^ Primary care diagnostic activity was then examined in the year pre‐diagnosis (GP visits, blood test use and abnormal blood test results) in LC and CRC patients. For analyses examining blood test use, the six RBCI, platelets and WBC were considered as one test type as they are all components of the full blood count (FBC) and normally requested together. Requests for the six APR test were analysed both combined as a composite variable and individually (FBC, inflammatory markers, ferritin and albumin) as they are not always ordered together. Estimates of the baseline characteristics and diagnostic activity were calculated for the full LC and CRC study cohorts, followed by the two subgroups of patients who were selected by their GP to have blood tests and who had any abnormal results in the year pre‐diagnosis.

Poisson regression modelling was used to examine trends over time in primary care diagnostic activity. For LC and CRC patients separately, the monthly rates of APR and FBC test requests were calculated for the 24 months pre‐diagnosis. Test request rate ratios (RR) were estimated comparing each monthly test request rate with the baseline testing rate at 24 months pre‐diagnosis. To identify the timing of the inflexion point when GP blood test requests first start to increase prior to cancer diagnosis we used the method previously described by Moullet et al. and Price et al.^11^, ^28^ This involved running a series of Poisson regression models with sequential monthly inflexion points and selecting the model with the best fit for estimating when testing rates first start to increase from the background rate. To assess the extent of blood test use over time, the monthly proportion of patients with an APR or FBC test were calculated for the 24‐months before diagnosis (plotting both the monthly incident percentage of patients tested, and the cumulative percentage tested over time starting from 24‐months pre‐diagnosis).

To examine trends over time in primary care blood test *abnormalities* in LC and CRC patients pre‐diagnosis, the above analyses were repeated replacing the binary blood test request variable (yes/no) with a blood test abnormality variable (yes/no). This was initially done using two composite measures for any RBCI abnormality or any APR abnormality, followed by separate models to examine abnormalities in each of the individual APR and RBCI test types. For the CRC cohort, sensitivity analyses were conducting excluding patients recorded as screen detected.

As anaemia is a well‐recognised alarm feature of cancer^29^, ^30^ that would often prompt urgent investigation or referral, in supplementary analyses we explored the potential added value of other blood test abnormalities for expediting cancer diagnosis. To do this we examined (a) the proportion of LC and CRC patients with blood test abnormalities occurring in the absence of/prior to anaemia (b) when abnormalities in anaemia and other blood test types were first detected prior to LC and CRC diagnosis.

## 3 RESULTS

The cohorts comprised 855 CRC patients and 399 LC patients with a new diagnosis of cancer during the study period. The CRC cohort was 57% male, with a mean age of 65 years and 31% of patients aged <60 years at diagnosis. The LC cohort was 60% male, with a mean age of 67 years and 24% aged <60 years at diagnosis (Table 1). Sensitivity analyses found no substantial differences between patient characteristics in the linked cohorts of CRC and LC patients when compared to patients in the ACCORD and AURORA datasets without linkage to primary care, or to CRC and LC patients in the Victorian Cancer Registry (Table S1). Patients with either cancer type visited their GP a mean of 6–7 times in the 6 months before cancer diagnosis and 10–12 times in the year leading up to diagnosis overall, with consultations being more frequent in patients who had blood tests requested and higher again in patients with one or more abnormal blood test results (Table 1).

**TABLE 1:** Baseline characteristics of lung and colorectal cancer patients who attend their GP, have a GP blood test and have an abnormal GP blood test result in the year pre‐diagnosis.

	CRC patients *N* = 855	CRC patients with a blood test *N* = 386	CRC patient with abnormal blood test *N* = 276	LC patients *N* = 399	LC patients with a blood test *N* = 242	LC patient with abnormal blood test *N* = 181
Male sex	490 (57%)	215 (56%)	155 (56%)	238 (60%)	146 (60%)	116 (64%)
Age at diagnosis						
20–29	7 (1%)	2 (0.5%)	2 (0.7%)	0 (0%)	0 (0%)	0 (0%)
30–39	34 (4%)	15 (4%)	9 (3%)	4 (1%)	2 (0.8%)	2 (1%)
40–49	73 (9%)	26 (7%)	20 (7%)	28 (7%)	13 (5%)	12 (7%)
50–59	154 (18%)	65 (17%)	41 (15%)	65 (16%)	38 (16%)	31 (17%)
60–69	250 (29%)	113 (29%)	71 (26%)	129 (32%)	81 (33%)	56 (31%)
70–79	201 (24%)	90 (23%)	68 (25%)	121 (30%)	76 (31%)	56 (31%)
80 and over	136 (16%)	75 (19%)	65 (24%)	52 (13%)	32 (13%)	24 (13%)
Mean (SD, range)	65 (13.6, 24–93)	66 (13.6, 24–93)	67 (14.0, 24–93)	67 (11.2, 32–93)	67 (10.7, 32–89)	67 (11.1, 32–88)
Median (IQR)	66 (56–75)	67 (57–77)	69 (59–79)	68 (60–75)	68 (61–75)	68 (60–75)
Year of diagnosis						
2001–2004	31 (4%)	4 (1%)	2 (0.7%)	0 (0%)	0 (0%)	0 (0%)
2005–2008	132 (15%)	22 (6%)	16 (6%)	6 (2%)	2 (0.8%)	1 (0.6%)
2009–2012	255 (30%)	96 (25%)	66 (24%)	73 (18%)	35 (14%)	25 (14%)
2013–2016	246 (29%)	137 (35%)	103 (37%)	175 (44%)	101 (42%)	85 (47%)
2017–2021	191 (22%)	127 (33%)	89 (32%)	145 (36%)	104 (43%)	70 (39%)
Active GP follow‐up time^a^						
Mean days (SD, range)	744 (387.8, 1–1095)	853 (330.6, 2–1095)	848 (336.2, 2–1095)	763 (389.5, 2–1095)	889 (313.2, 10–1095)	901 (303.3, 29–1095)
Median days (IQR)	962 (346–1070)	1030 (703–1078)	1033 (697–1079)	994 (352–1078)	1051 (841–1083)	1057 (877–1082)
Number of GP visits in 6 m pre‐diagnosis						
0	89 (10%)	14 (4%)	8 (3%)	54 (14%)	17 (7%)	12 (7%)
1–3	249 (29%)	79 (20%)	55 (20%)	88 (22%)	36 (15%)	21 (12%)
4–6	227 (27%)	109 (28%)	63 (23%)	73 (18%)	45 (19%)	32 (18%)
7–9	123 (14%)	79 (20%)	63 (23%)	65 (16%)	46 (19%)	32 (18%)
≥ 10	167 (20%)	105 (27%)	87 (32%)	119 (30%)	98 (41%)	84 (46%)
Mean (SD, range)	6 (5.3, 0–34)	7 (5.5, 0–34)	8 (5.8, 0–34)	7 (6.8, 0–41)	9 (7.0, 0–41)	10 (7.1, 0–41)
Median (IQR)	5 (2–8)	6 (4–10)	7 (4–10)	6 (1–11)	8 (4–13)	9 (5–13)
Number of GP visits in 12 m pre‐diagnosis						
1–3	226 (26%)	43 (11%)	24 (9%)	111 (28%)	31 (13%)	18 (10%)
4–6	157 (18%)	64 (17%)	40 (14%)	47 (12%)	28 (12%)	19 (11%)
7–9	136 (16%)	72 (19%)	53 (19%)	52 (13%)	33 (14%)	23 (13%)
≥ 10	336 (39%)	207 (54%)	159 (58%)	189 (47%)	150 (62%)	121 (67%)
Mean (SD)	10 (8.7, 1–60)	12 (9.1, 1–60)	13 (9.5, 1–60)	12 (10.7, 1–70)	18 (11.3, 1–70)	16 (11.7, 1–70)
Median (IQR)	7 (3–14)	10 (6–17)	11 (7–18)	9 (3–16)	13 (7–21)	14 (8–21)
Stage						
1	174 (20%)	80 (21%)	53 (19%)	86 (22%)	57 (24%)	41 (23%)
2	224 (26%)	91 (24%)	65 (24%)	32 (8%)	18 (7%)	14 (8%)
3	196 (23%)	89 (23%)	61 (22%)	105 (26%)	63 (26%)	51 (28%)
4	152 (18%)	74 (19%)	57 (21%)	161 (40%)	94 (39%)	69 (38%)
Missing	109 (13%)	52 (13%)	40 (14%)	15 (4%)	10 (4%)	6 (3%)

Abbreviations: CRC, colorectal cancer; IQR, Interquartile range; LC, lung cancer; SD, standard deviation.

^a^ Time from first GP encounter in the 2 years before diagnosis until cancer date (patients must have at least one encounter per year to be considered active).

### 3.1 Tumour characteristics

For CRC patients, 41% were diagnosed at a late stage (Stage 3 or 4), with one third of tumours located in the rectum (38%) and two thirds in the colon (62%). Fifteen per cent of CRC patients were recorded as screen detected (from data in the hospital registry), 79% were diagnosed after presenting with symptoms, 2% were incidental diagnoses and data on route to diagnosis were missing for 4% (Table S2). For LC patients, 66% were diagnosed at a late stage. Sixty‐eight per cent of patients were diagnosed symptomatically and 24% were incidental findings (Table S2). Thirty‐six per cent of LC patients had a respiratory co‐morbidity recorded in the hospital cancer registry and 81% were current or past smokers (Table S2). For both cancers, stage distribution was comparable between patients in the full study cohort, those selected for blood testing and those who had abnormal blood test results (Table 1).

### 3.2 Blood test request

For CRC patients, over a third had a GP requested APR (38%) or RBCI (39%) blood test requested in the year before cancer diagnosis (Table 2; Figure S1). The monthly rate of GP blood test requests started to increase from 7 months before CRC diagnosis (inflexion point) (Figure S2; Table S3). FBC was the most commonly requested blood test (Table 2) and ferritin had the largest increase in requests from baseline (at −24 months), increasing 7‐fold (FBC, LFT and inflammatory marker requests increased 4‐fold) (Figure S2).

**TABLE 2:** Association between cancer diagnosis and GP blood test use and abnormal results in the year preceding diagnosis.

Blood test request in the 12 months before cancer diagnosis		
Blood test	Colorectal cancer patients (*n* = 855)	Lung cancer patients (*n* = 399)
Any APR test	327 (38%)	198 (50%)
Platelet	306 (36%)	193 (48%)
Albumin	311 (36%)	191 (48%)
Inflammatory marker (ESR/CRP)	123 (14%)	113 (28%)
Ferritin	191 (22%)	82 (21%)
Total WBC count	307 (36%)	161 (40%)
Any RBCI test	334 (39%)	204 (51%)
Haemoglobin	334 (39%)	203 (51%)
HCT	263 (31%)	184 (46%)
MCHC	232 (27%)	163 (41%)
MCV	306 (36%)	184 (46%)
RBC count	297 (35%)	191 (48%)
RBCDW	213 (25%)	161 (40%)
Abnormal blood test results in the 12 months before cancer diagnosis		
Any abnormal APR test^a^	177 (54%)	117 (59%)
Raised platelet^a^	43 (14%)	34 (18%)
Low albumin^a^	22 (7%)	24 (13%)
Raised inflammatory marker (ESR/CRP)^a^	84 (68%)	84 (74%)
Raised ferritin^a^	22 (12%)	17 (21%)
Low ferritin^a^	89 (47%)	11 (13%)
Raised total WBC count^a^	43 (14%)	38 (24%)
Any abnormal RBCI test^a^	209 (63%)	124 (61%)
Low haemoglobin^a^	140 (42%)	43 (21%)
Low HCT^a^	110 (42%)	41 (22%)
Low MCHC^a^	39 (17%)	8 (5%)
Low MCV^a^	58 (19%)	13 (7%)
Low RBC count^a^	89 (30%)	55 (29%)
Raised RBCDW^a^	140 (66%)	96 (60%)

Abbreviations: APR, acute phase reactant; CRP, c‐reactive protein; ESR, erythrocyte sedimentation rate; HCT, haematocrit; MCHC, mean cell haemoglobin concentration; MCV, mean cell volume; RBC, red blood cell; RBCDW, red blood cell distribution width; RBCI, red blood cell indices; WBC, white blood cell.

^a^ Percentage out of all patients who had a test result.

For LC patients, half had an APR (50%) or RBCI (51%) test requested pre‐diagnosis (Table 2; Figure S1), with GP blood requests increasing from 6 months pre‐diagnosis (Figure S2; Table S3). FBC was the most requested test (Table 2) and inflammatory marker and LFT requests increased the most (3‐fold) from baseline (FBC and ferritin increased 2‐fold) (Figure 1) (Figure S2).

### 3.3 Abnormal test results

#### 3.3.1 Proportion of patients with abnormal results

Among tested CRC patients, 54% had at least one abnormal APR result and 63% had an abnormal RBCI result in the 12 months before diagnosis. Inflammatory marker tests and RBCDW had the highest percentage of abnormal results in the year pre‐diagnosis (68% of patients tested had raised inflammatory markers and 66% had raised RBCDW). In terms of blood test markers of iron deficiency anaemia, 47% of tested patients had low ferritin and 42% had anaemia (Table 2). Sensitivity analyses found no substantial differences in the proportion of CRC patients with a blood test or with an abnormal result when excluding the 126 patients (15%) who were recorded as screen detected (Table S4). For both APR and RBCI tests, the monthly proportion of CRC patients with at least one abnormal result progressively increased from 9 months before diagnosis from a baseline of <3% of patients to 9%–10% in the month preceding diagnosis (Figure S3).

Among tested LC patients, 59% had one or more abnormal APR test results and 61% had an abnormal RBCI test in the year pre‐diagnosis. The blood tests most commonly abnormal in tested LC patients pre‐diagnosis were raised inflammatory markers (68% of patients tested had an abnormal result) and raised RBCDW (60%), followed by raised RBC count (29%). 21% of patients tested had anaemia (Table 2). The proportion of patients with an abnormal APR or RBCI result progressively increased from 4 months before diagnosis from a slightly higher baseline rate of <5%–12%–13% (Figure S3).

#### 3.3.2 Trends over time in blood test abnormalities before cancer diagnosis

**FIGURE 2:**
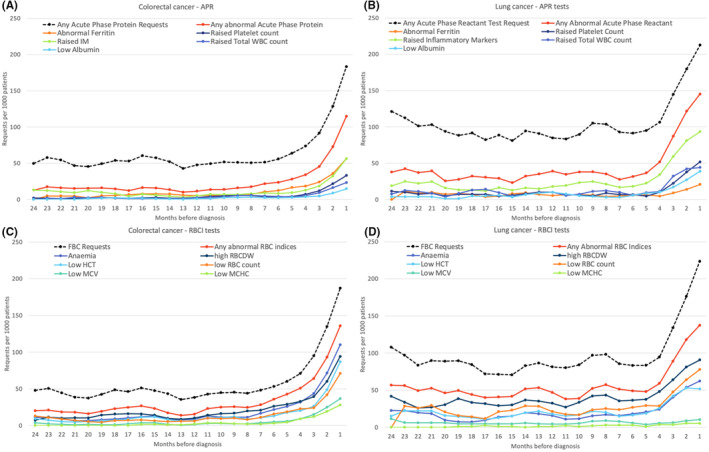
Monthly rates of abnormal GP blood tests in colorectal cancer (panels A and C) and lung cancer patients (panels B and D) in the 24 months before diagnosis/index date (3 month moving average) Dashed line represents the monthly rate of patients tested. APR, acute phase reactant; FBC, full blood count; HCT, haematocrit; IM, inflammatory marker; MCHC, mean cell haemoglobin concentration; MCV, mean cell volume; RBC, red blood cell; RBCDW, red blood cell distribution width; RBCI, red blood cell index; WBC, white blood cell.

In CRC patients, the rate of blood test abnormalities started to increase from 8 months before cancer diagnosis. Abnormalities in one or more APR test results increased from a baseline rate of 13 abnormal tests per 1000 patients/month up to 115 per 1000 patients/month in the month preceding diagnosis. Similar increases were seen for RBCI abnormalities (rate of any RBCI abnormality increased from 20 to 136 per 1000 patients/month) (Figure 2).

In LC patients, the rate of blood test abnormalities started to increase from before diagnosis, with the baseline rate of abnormal APR or RBCI tests rising from 38 and 57 per 1000 patients/month, respectively, to 145 and 136 abnormal results per 1000 patients/month in the month preceding diagnosis (Figure 2).

#### 3.3.3 Blood test abnormality signature for CRC

In CRC patients, the highest rates of APR abnormalities pre‐diagnosis were for abnormal ferritin and raised inflammatory markers, which both increased from a baseline rate of <5 abnormal tests per 1000 patients/month to 56 and 33 per 1000 patients/month, respectively. Increases in all six RBCI abnormalities were observed prior to CRC diagnosis, with the most common being high RBCDW and anaemia, which increased from a baseline rate of 7–11 abnormal tests per 1000 patients/month up to 94–110 tests per 1000 patients/month immediately before CRC diagnosis, respectively. The largest increases in abnormal results were for raised platelets (16‐fold increase), abnormal ferritin (15‐fold increase) and raised RBCDW (13‐fold increase) (Figure 2).

#### 3.3.4 Blood test abnormality signature for LC

Among LC patients, the highest rate of APR abnormalities pre‐diagnosis was raised inflammatory markers followed by raised platelets. Raised inflammatory markers increased from a baseline rate of 19–93 abnormal results per 1000 patients/month and raised platelets rose from 11 to 52 abnormal results per 1000 patients/month in the month before LC diagnosis. Of the six RBCI, pre‐diagnostic increases in abnormalities were seen in RBCDW, haemoglobin, RBC count and HCT, with no increases observed in MCHC or MCV abnormalities. The most common RBCI abnormalities were raised RBCDW and low RBC count, which both occurred more frequently before LC diagnosis than anaemia. Rates of raised RBCDW increased from 42 abnormal tests per 1000 patients/month to 91 per 1000 patients/month, and low RBC count increased from 26 to 78 abnormal tests per 1000 patients/month. The largest pre‐diagnostic increases in blood test abnormalities were for low albumin and raised platelets, which both increased 8‐fold, followed by raised total WBC count (6‐fold increase) (Figure 2).

#### 3.3.5 Abnormal blood tests occurring before or in the absence of anaemia

**FIGURE 3:**
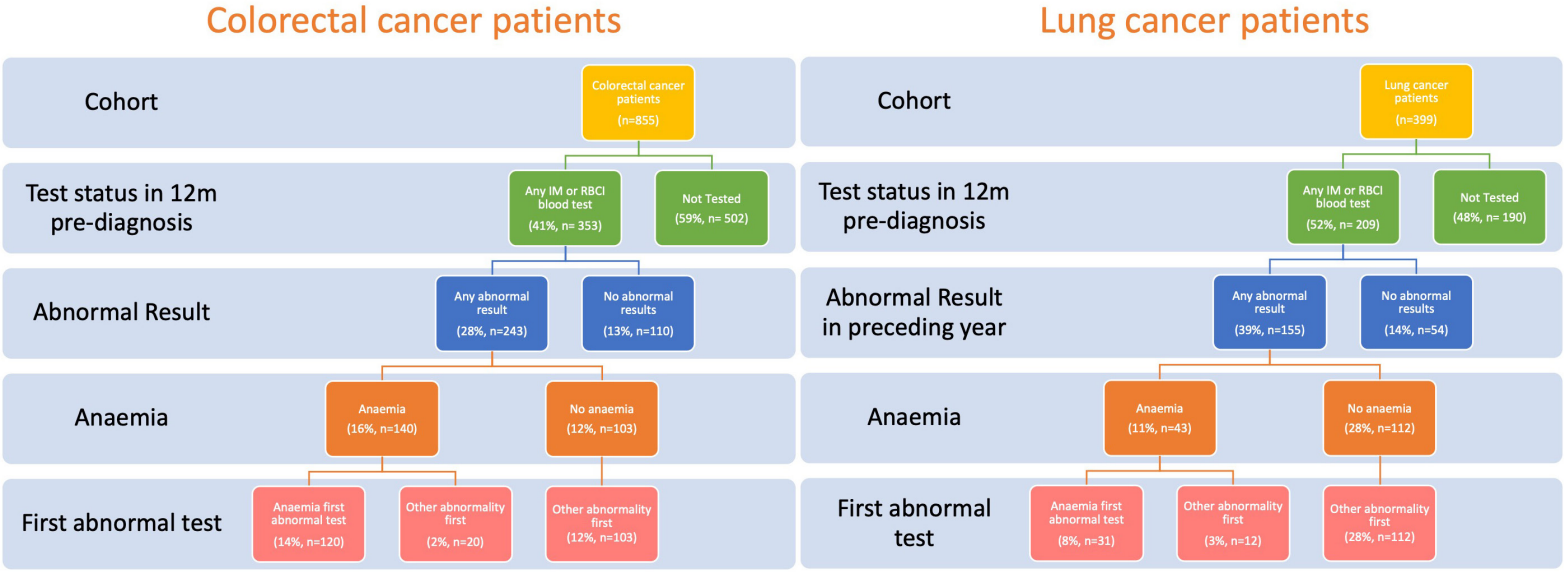
Percentage of colorectal and lung cancer patients with abnormal blood test results detected before or in the absence of anaemia.

Of the 243 CRC patients with an abnormal blood test result in the year pre‐diagnosis, over half had another blood test abnormality detected before (or in the absence of) anaemia: 103 patients had no anaemia and 20 patients had another abnormality first. In LC patients, 155 had an abnormal blood result and, in 124 of these patients anaemia was not the first abnormality detected (Figure 3).

#### 3.3.6 Timing of first abnormal blood result before cancer diagnosis

**FIGURE 4:**
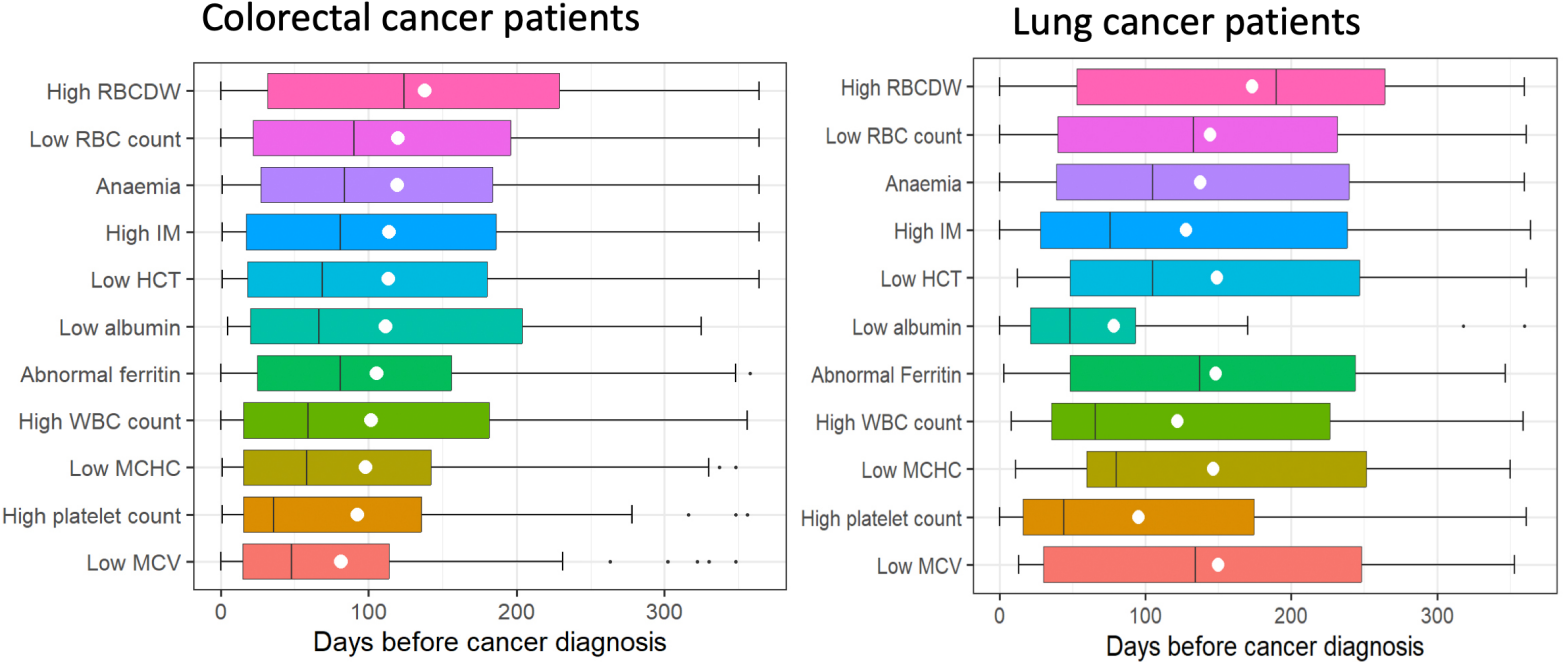
Timing of first abnormal test before colorectal and lung cancer diagnosis. Box and whisker plot shows median and interquartile range; white dot represents mean; HCT, haematocrit; IM, inflammatory marker; MCHC, mean cell haemoglobin concentration; MCV, mean cell volume; RBC, red blood cell; RBCDW, red blood cell distribution width; WBC, white blood cell.

For both CRC and LC patients the earliest blood test abnormality detected on average was RBCDW, which occurred a median of 124 days before CRC diagnosis (IQR 32–229) and 190 days before LC diagnosis (IQR 53–264). This was on average earlier than anaemia, which occurred a median of 84 days pre‐diagnosis in CRC and 105 days pre‐diagnosis in LC (Table 3; Figure 4).

**TABLE 3:** Average number of days before cancer diagnosis when first abnormal GP blood test (acute phase reactant or red blood cell index) was detected.

Timing of first abnormal test									
Blood test	Colorectal cancer patients				Lung cancer patients				*p* value
	*N*	Median (IQR)	Mean (SD)	Range	*N*	Median (IQR)	Mean (SD)	Range	
Red blood cell index tests									
Any RBCI test abnormality	209	121 (34–231)	141 (111.9)	1–364	124	165 (46–257)	160 (116.6)	0–361	0.14
Anaemia	140	84 (27–186)	120 (109.0)	1–364	43	105 (35–242)	138 (113.1)	0–360	0.34
Raised RBCDW	140	124 (32–229)	138 (112.4)	0–364	96	190 (53–264)	173 (113.9)	0–360	0.02
Low MCV	58	48 (15–115)	81 (91.0)	0–348	13	134 (30–248)	150 (122.4)	13–353	0.02
Low HCT	110	69 (18–180)	113 (110.2)	1–364	41	105 (48–247)	149 (114.0)	12–361	0.08
Low RBC count	89	90 (22–196)	120 (110.7)	0–364	55	133 (36–234)	144 (114.5)	0–361	0.21
Low MCHC	39	58 (15–150)	98 (107.7)	1–348	8	80 (59–266)	146 (127.8)	11–350	0.27
Acute phase protein blood tests									
Any APR test abnormality	177	82 (25–185)	115 (106.1)	0–364	117	103 (30–254)	141 (118.2)	0–364	0.05
Abnormal ferritin conc.	109	81 (25–156)	105 (98.3)	0–358	27	137 (44–250)	148 (116.2)	3–347	0.05
Raise platelet count	43	36 (15–137)	92 (108.6)	1–356	34	44 (16–187)	95 (113.3)	0–361	0.91
Low albumin	22	67 (20–216)	111 (106.4)	5–325	24	48 (20–95)	78 (92.6)	0–360	0.27
Raised inflammatory marker (ESR/CRP)	84	81 (17–188)	114 (109.8)	1–364	84	76 (28–242)	128 (116.4)	0–364	0.42
Raised WBC count	43	59 (15–185)	102 (106.8)	0–356	38	66 (35–230)	122 (108.6)	8–359	0.41

Abbreviations: APR, acute phase reactant; conc, concentration; CRP, c‐reactive protein; ESR, erythrocyte sedimentation rate; HCT, haematocrit; IM, inflammatory marker; IQR, interquartile range; MCHC, mean cell haemoglobin concentration; MCV, mean cell volume; RBC, red blood cell; RBCDW, red blood cell distribution width; RBCI, red blood cell indices; SD, standard deviation; WBC, white blood cell.

*Note*: Starting from 12 months before cancer diagnosis in patients with an abnormal result; colorectal cancer *n* = 243, lung cancer *n* = 155. *p* value from *t*‐test comparing means in colorectal cancer patients with lung cancer patients.

## 4 DISCUSSION

### 4.1 Key findings

GP blood test requests increase from 7 months before CRC diagnosis and 6 months before LC diagnosis. This is the first study of its kind examining primary care diagnostic activity prior to cancer diagnosis using linked Australian primary care data. It indicates that an appreciable diagnostic window exists in the Australian setting where there is increased GP healthcare use by patients several months before cancer diagnosis and potential opportunities exist for earlier detection. In the year before diagnosis, over half of LC and CRC patients tested have an abnormal APR blood test result and two thirds had an abnormal RBCI test. The rates of these blood test abnormalities increase several months before cancer diagnosis and they are frequently detected before or in the absence of anaemia. This suggests they are early signals of as‐yet‐undetected cancer. CRC and LC patients have distinct signatures of blood test abnormalities pre‐diagnosis. These blood test abnormalities have potential for helping to identify patients in need of urgent investigation for different possible cancer sites, especially in patients consulting with non‐specific symptoms.

### 4.2 Comparison with the literature

#### 4.2.1 Length of the diagnostic window before LC and CRC diagnosis

The diagnostic window describes the time point when healthcare activity first starts to increase prior to diagnosis and represents the maximum length of time diagnosis could potentially be brought forward.^7^ The use of linked GP EHR to estimate the diagnostic window length before cancer is well established in the literature.^31^ Previous findings for LC and CRC have been based on UK,^11^, ^12^, ^32^, ^33^ Danish,^7^, ^9^, ^10^, ^34^ Dutch^35^ and Swedish^36^ studies, which indicate that patterns of healthcare use and diagnostic window lengths vary between countries and by cancer type.^31^ One Australian study^37^ has examined this phenomenon using GP billing data from patients in the 45 and up cohort.^38^, ^39^ It compared the monthly odds of having a GP consultation in cancer patients versus controls and found that consultations were more likely in cancer patients from 4 months before LC and CRC diagnosis. The study only had an 18% response rate and participants were not representative of the Australian population as the study intentionally oversampled patients aged ≥80 years and those living in rural areas, and excluded patients with low English literacy. In the few international studies of consultation patterns before cancer diagnosis, there was wide variation in the diagnostic window length: Increases in GP consultations have been reported from 3 to 24 months before CRC diagnosis^7^, ^32^, ^34^, ^35^, ^36^, ^40^, ^41^, ^42^ and 3–6 months before LC diagnosis.^10^, ^32^, ^33^, ^34^, ^36^ Few studies have used other forms of GP healthcare use (beyond consultations) to estimate the diagnostic window length for LC and CRC. These have reported increases in GP prescribing 6–18 months before CRC diagnosis^7^, ^9^, ^35^ and 4–6 months before LC diagnosis^9^, ^10^; increases in GP imaging and lung function tests in the year before LC diagnosis^10^; and increases in haemoglobin requests 17 months prior to CRC diagnosis.^7^ Our study addressed an important gap in the literature by examining GP healthcare use before LC and CRC diagnosis using linked Australian GP data. By examining four types of GP blood test requests, we demonstrate a diagnostic window up to 7 months before CRC and 6 months before LC diagnosis. This indicates similar opportunities to expedite CRC and LC diagnosis potentially exist in Australian primary care if these patients can be identified and investigated/referred earlier in the diagnostic window.

#### 4.2.2 Blood test abnormalities as potential early signals of cancer

Two UK proof‐of‐concept studies have examined trends in blood test abnormalities prior to LC and CRC diagnosis.^11^, ^12^ McDonald et al. compared mean blood test levels over time pre‐diagnosis in LC patients and controls, finding increased CRP, platelet count and WBC levels from 6 months pre‐diagnosis.^12^ Similarly, Moullet et al. found that rates of anaemia, raised platelets and raised inflammatory markers increased from 9 months prior to CRC diagnosis.^11^ Our study confirmed these findings in an Australian dataset, demonstrating increases in the rate of raised inflammatory markers, total WBCs and platelets from 6 months before LC diagnosis and increased rates of anaemia, raised platelets and inflammatory markers from 8 months before CRC diagnosis. We expanded on past findings by showing pre‐diagnostic increases in the rate of additional APR abnormalities (ferritin and low albumin) and RBCI abnormalities (raised RBCDW, low RBC count, HCT, MCHC and MCV). The importance of many of these abnormalities as early signals of cancer has not been previously examined, with previous studies showing an association between increased RBCDW and LC and CRC, indicating possible diagnostic value, but not examining when abnormalities are first detectable before cancer diagnosis in primary care.^43^, ^44^, ^45^ The findings from this study, especially in relation to RCBI abnormalities such as raised RBCDW which may be less likely to prompt investigation in the absence of anaemia, identify additional flags of undiagnosed cancer in general practice which are often detected several months before cancer diagnosis. Additionally, we identified distinct pre‐diagnostic signature patterns in blood test abnormalities between LC and CRC patients and report the average timing of the first detectable blood test abnormalities before cancer diagnosis—demonstrating that many abnormalities occur before or in the absence of anaemia and have potential for expediting cancer diagnosis.

#### 4.2.3 Strengths and weaknesses

As far as the authors are aware, no previous study has examined patterns in GP blood tests over time prior to cancer diagnosis in the Australian setting. Australia has higher rates of blood testing and lower thresholds for ordering GP investigations than other countries, which could present earlier opportunities for detecting blood test abnormalities and associated cancer in patients.^46^, ^47^ Use of two primary care datasets is an important strength as general practice is where most cancer patients first present, and this ensured a large, representative population for linkage. We examined 12 different tests, providing a comprehensive evaluation of GP blood tests prior to cancer diagnosis, using statistical estimation of inflexion points and comparing patterns to baseline rates and between LC and CRC patients to identify distinct signatures. GP blood results are electronically transmitted into EHRs from laboratories, which increases accuracy and completeness. Results were categorised as normal or abnormal, but further research examining the magnitude of abnormality and considering borderline normal results could reveal additional associations. Data on the indication for test requests, use of faecal occult blood testing (iFOBT) and timing of referrals for specialist diagnostic tests like colonoscopy were not available. Future work exploring these factors and co‐occurring symptoms could help identify patient groups most likely to benefit from the study findings and areas of potential diagnostic delay. Although Victorian Cancer Registry and cancer screening data were not available, the hospital cancer registries used in this study contain detailed, clinician recorded cancer data from multiple hospitals receiving referrals from across Victoria. Increased access to additional datasets, with mechanisms to systematically link them to existing data sources, would provide a more complete clinical picture to support future early diagnosis research and health system improvement. Most patients are likely metropolitan based, which could limit the generalisability of findings to rural areas where healthcare access may differ. In Australia patients are not limited to seeing a GP at a single practice, meaning GP encounters at practices not included in the primary care datasets will be missed. To counter this, we only included patients who had visited a GP in that practice in the same year and a recent study showed that 90% of Australians attend a regular general practice.^48^

#### 4.2.4 Implications

There is an increase in primary care diagnostic activity from 7 months before CRC diagnosis and 6 months before LC diagnosis. This indicates that some patients with as‐yet‐undetected cancer are presenting to their GP several months before their cancer is diagnosed with symptoms prompting blood test investigation. This diagnostic window contains potential opportunities for earlier LC and CRC diagnosis in Australian primary care. As blood test use is common this would need to be supported by diagnostic advances, such as computer decision support,^49^ novel circulating tumour DNA tests^50^ and optimising information from existing common blood test results,^51^, ^52^ to help identify these patients and prompt earlier investigation to detect cancer.

Abnormal results in many commonly used blood tests increase several months before diagnosis and are early markers of LC and CRC. These abnormalities frequently occur in patients who do not have anaemia or occur before anaemia is detectable. They therefore could provide added value in diagnosing cancer earlier in some patients, particularly in those without alarm symptoms that would already prompt further investigation or referral. Many RBCI become abnormal up to several months before the haemoglobin, such as raised RBCDW, low RBC count and low HCT, suggesting that GPs should pay greater attention to these additional measures reported in a FBC result and not just the haemoglobin and MCV. This is particularly relevant as less than half of the CRC patients in this study had anaemia or low ferritin prior to diagnosis. We found distinct patterns in the types of blood test abnormalities occurring pre‐diagnosis in patients with as‐yet‐undetected LC and CRC, which could help to differentiate cancers of different sites in patients with non‐specific cancer symptoms. It is important to note that these blood test abnormalities are common in general practice and can be markers of many alternative diagnoses, such as infection or other inflammatory conditions. A single abnormal blood test result will likely have low predictive value and low specificity for detecting underlying cancer in isolation. Therefore, to understand the value of these blood test abnormalities in improving cancer diagnosis, further studies are required examining their predictive value for detecting underlying cancer by age and sex in patients with and without cancer presenting symptomatically to primary care, including consideration of blood test result combinations, magnitude and trends, and alongside other pre‐diagnostic features recorded in the GP EHR (symptoms, prescriptions and demographics). This would help determine in which circumstances these abnormalities would indicate high risk of underlying cancer warranting further specialist cancer investigation or referral.

Finally, we found that one third of CRC patients and half of LC patients did not have a GP requested blood test in the year before their cancer diagnosis. These findings are similar to findings reported by Cranfield et al. from a UK study.^53^ It is possible that increasing GP blood test requests in some of these patients could support earlier cancer diagnosis, but this would first require empirical evidence on cancer risk for different abnormal blood test results and guidelines on which patients with such abnormalities should be considered for further specialist investigations. It should be noted that any increase in primary care test use will reduce the predictive value of blood test abnormalities for detecting underlying cancer, due to the lower pre‐test probability of cancer in this less selective population.^54^

## 5 CONCLUSION

We have shown that in the Australian context, many patients with as‐yet‐undetected LC and CRC are presenting to primary care several months before diagnosis with symptoms prompting blood test investigation by their GP. This evidence from a flexible healthcare system with high rates of blood testing, ease of GP access to rapid investigations and free movement of patients between public and private systems^46^, ^47^ mirrors that observed in European health systems. Cancer diagnosis could be potentially brought forward up to 6 months for LC and 7 months for CRC in some patients if diagnostic technologies can be developed and deployed to help identify patients at higher risk of underlying cancer and prompt further diagnostic investigation. We identified blood test abnormalities in APRs and RBCI which commonly occur several months before cancer diagnosis and are early signals of LC and CRC in tested patients that could help distinguish between cancer sites. Different blood test abnormalities are first detectable at different times pre‐diagnosis, with many of them, particularly certain RBCI, occurring either in the absence of anaemia or considerably before anaemia is detected in many patients. If data from these blood tests can be combined with other pre‐diagnostic information, they could support GPs to prioritise patients for investigation to expedite cancer diagnosis in primary care.

## AUTHOR CONTRIBUTIONS

**Meena Rafiq:** Conceptualization (lead); data curation (lead); formal analysis (lead); investigation (lead); methodology (lead); writing – original draft (lead); writing – review and editing (equal). **Allison Drosdowsky:** Data curation (supporting); formal analysis (supporting); methodology (supporting); writing – review and editing (equal). **Ben Solomon:** Data curation (supporting); methodology (supporting); resources (equal); writing – original draft (supporting); writing – review and editing (equal). **Marliese Alexander:** Data curation (supporting); methodology (supporting); resources (equal); writing – review and editing (supporting). **Peter Gibbs:** Data curation (supporting); resources (equal); writing – review and editing (equal). **Gavin Wright:** Data curation (supporting); investigation (supporting); resources (equal); writing – review and editing (equal). **Justin M. Yeung:** Data curation (supporting); resources (equal); writing – review and editing (equal). **Georgios Lyratzopoulos:** Conceptualization (supporting); methodology (supporting); supervision (supporting); writing – original draft (supporting); writing – review and editing (equal). **Jon Emery:** Conceptualization (supporting); formal analysis (supporting); funding acquisition (lead); methodology (supporting); resources (equal); supervision (lead); writing – original draft (supporting); writing – review and editing (equal).

## FUNDING INFORMATION

MR and the work presented in this paper were supported by Victorian Comprehensive Cancer Centre (VCCC) strategic research funding. JE is supported by an NHMRC Investigator grant (APP1195302). The study aligns to (but was not directly supported by) the RREDD‐EHR project supported by the International Alliance for Cancer Early Detection, Cancer Research UK (C18081/A31373). GL acknowledges an Advanced Clinician Scientist Fellowship from Cancer Research UK (C18081/A18180).

## CONFLICT OF INTEREST STATEMENT

None of the authors have any conflicts of interest to declare.

## ETHICS STATEMENT

The protocol for this project was approved by the Melbourne Health Human Research Ethics committee (protocol number: 202005/8 and 202003/8) and has MedicineInsight Data Governance approval (*approval number: 2016‐014)*. This research used de‐identified patient data from the Patron primary care data repository (extracted from consenting general practices), that has been created and is operated by the Department of General Practice and Primary Care, The University of Melbourne: www.gp.unimelb.edu.au/datafordecisions and MedicineInsight dataset (extracted from consenting general practices), that is operated by the Australian Commission on Safety and Quality in Health Care. Consistent with National Health and Medical Research Council (NHMRC) ethical guidelines for the use of health‐related data, patients are not required to give written consent due to the non‐identifiable nature of the data collected. This process has been approved by the Royal Australian College of General Practitioners (RACGP) ethics committee.

## Supporting information


Data S1.

## Data Availability

The data that support the findings of this study are available from the VCCC Alliance Data Connect. Restrictions apply to the availability of these data, which were used under approvals for this study. Data are available from https://vcccalliance.org.au/our‐work/research‐and‐translation/data‐connect/how‐to‐access‐the‐hub/ with the permission of BioGrid.
