# Supplementary material for: Trends in primary care blood tests prior to lung and colorectal cancer diagnosis—A retrospective cohort study using linked Australian data
Source: Cancer Med. 2024 Jul 13;13(14):e70006. doi: 10.1002/cam4.70006 (PMC11245636; doi:10.1002/cam4.70006)
Supplement: Supplementary file 1 — Data S1. [file CAM4-13-e70006-s001.docx]

## Supplementary data

##### Supplementary Table S1: Baseline characteristics of linked and unlinked lung and colorectal cancer patients from the ACCORD and AURORA cohorts and from all lung and colorectal cancer patients in the Victorian Cancer Registry

*For patients reported with a new diagnosis of colorectal cancer in the Victorian Cancer Registry in 2016. SD, standard deviation; IQR, Interquartile range; P value from Chi2 test comparing lung cancer and colorectal cancer cohorts *time from first GP encounter in the 2 years before diagnosis until cancer date (patients must have at least one encounter per year to be considered active).

|  | **Linked CRC patients**  **N=855** | **Unlinked CRC patients**  **N=6,571** | **State-wide CRC patients***  **N=3,863** | **Linked LC patients**  **N=399** | **Unlinked LC patients**  **N=2,977** | **State-wide LC patients***  **N=2,949** |
| --- | --- | --- | --- | --- | --- | --- |
| Male sex | 491 (57%) | 3,804 (58%) | 2,130 (55%) | 238 (60%) | 1,711 (57%) | 1,630 (55%) |
| Age at diagnosis |  |  |  |  |  |  |
| <20 | 0 (0%) | 8 (0.1%) | 24 (0.6%) | 0 (0%) | 1 (0%) | 1 (0%) |
| 20–29 | 7 (1%) | 55 (0.8%) | 36 (0.9%) | 0 (0%) | 16 (0.5%) | 6 (0.2%) |
| 30–39 | 34 (4%) | 195 (3%) | 100 (3%) | 4 (1%) | 48 (2%) | 22 (0.7%) |
| 40–49 | 73 (9%) | 494 (8%) | 235 (6%) | 28 (7%) | 178 (6%) | 77 (3%) |
| 50–59 | 154 (18%) | 1,156 (18%) | 524 (14%) | 65 (16%) | 529 (18%) | 347 (12%) |
| 60–69 | 250 (29%) | 1,857 (28%) | 860 (22%) | 129 (32%) | 992 (33%) | 751 (25%) |
| 70–79 | 201 (24%) | 1,841 (28%) | 1,157 (30%) | 121 (30%) | 902 (30%) | 1,015 (34%) |
| 80 and over | 136 (16%) | 965 (15%) | 927 (24%) | 52 (13%) | 311 (10%) | 730 (25%) |
| Mean (SD, range) | 65 (13.6, 24-93) | 66 (13.1, 15-100) |  | 67 (11.2, 32-93) | 66 (11.3, 19-94) |  |
| Median (IQR) | 66 (56-75) | 67 (58-76) |  | 68 (60-75) | 67 (59-74) |  |
| Year of diagnosis |  |  |  |  |  |  |
| Pre 2000 | 0 (0%) | 726 (11%) |  | 0 (0%) | 15 (0.5%) |  |
| 2001–2004 | 31 (4%) | 862 (13%) |  | 0 (0%) | 12 (0.4%) |  |
| 2005–2008 | 132 (15%) | 1,205 (18%) |  | 6 (2%) | 50 (2%) |  |
| 2009–2012 | 255 (30%) | 1,395 (21%) |  | 73 (18%) | 510 (17%) |  |
| 2013–2016 | 246 (29%) | 1,282 (20%) |  | 175 (44%) | 1,206 (41%) |  |
| 2017–2021 | 191 (22%) | 1,101 (17%) |  | 145 (36%) | 1,184 (40%) |  |

##### Supplementary Table S2: Tumour characteristics of lung and colorectal cancer patients from the clinical registry

| **Characteristic** | **Colorectal cancer patients**  **N= 855** | **Characteristic** | **Lung cancer patients**  **N= 399** |
| --- | --- | --- | --- |
| Stage |  | Stage |  |
| 1 | 174 (20%) | 1 | 86 (22%) |
| 2 | 224 (26%) | 2 | 32 (8%) |
| 3 | 196 (23%) | 3 | 105 (26%) |
| 4 | 152 (18%) | 4 | 161 (40%) |
| Missing | 109 (13%) | Missing | 15 (4%) |
| Diagnosis route |  | Diagnosis route |  |
| Incidental | 21 (2%) | Incidental | 96 (24%) |
| Screen detected | 126 (15%) | Symptomatic | 271 (68%) |
| Symptomatic | 675 (79%) | Missing | 32 (8%) |
| Missing | 33 (4%) | Tumour Morphology |  |
| Tumour Morphology |  | Adenocarcinoma | 212 (53%) |
| Adenocarcinoma | 744 (87%) | Squamous cell | 88 (22%) |
| Other | 10 (1%) | Small cell | 35 (9%) |
| Missing | 101 (12%) | Non-small cell NOS | 35 (9%) |
| Site |  | Mesothelioma | 9 (2%) |
| Colon | 529 (62%) | Other | 17 (4%) |
| Rectum | 326 (38%) | Missing | 3 (1%) |
| Inflammatory bowel disease |  | Respiratory co-morbidity |  |
| Yes | 15 (2%) | Yes | 144 (36%) |
| No | 803 (94%) | No | 228 (57%) |
| Missing | 37 (4%) | Missing | 27 (7%) |
| Tobacco History |  | Tobacco History |  |
| Current | 128 (15%) | Current | 82 (21%) |
| Past | 237 (28%) | Past | 238 (60%) |
| Never | 431 (50%) | Never | 61 (15%) |
| Missing | 59 (7%) | Missing | 18 (5%) |
|  |  | Tobacco Pack Years |  |
|  |  | 0 | 64 (16%) |
|  |  | 1-19 | 52 (13%) |
|  |  | 20-39 | 73 (18%) |
|  |  | 40-59 | 96 (24%) |
|  |  | 60-79 | 41 (10%) |
|  |  | ≥80 | 40 (10%) |
|  |  | Missing | 33 (8%) |
|  |  | Mean (SD, range) | 39 (34.2, 0-198) |

##### Supplementary Figure S1: Monthly with a GP blood test request for the proportion of colorectal and lung cancer patients 24 months before diagnosis date

Panels A and B display incident percentages (3 month moving average) and panels C and D display cumulative percentages over time. FBC, full blood count, LFT, liver function test; IM, inflammatory marker.


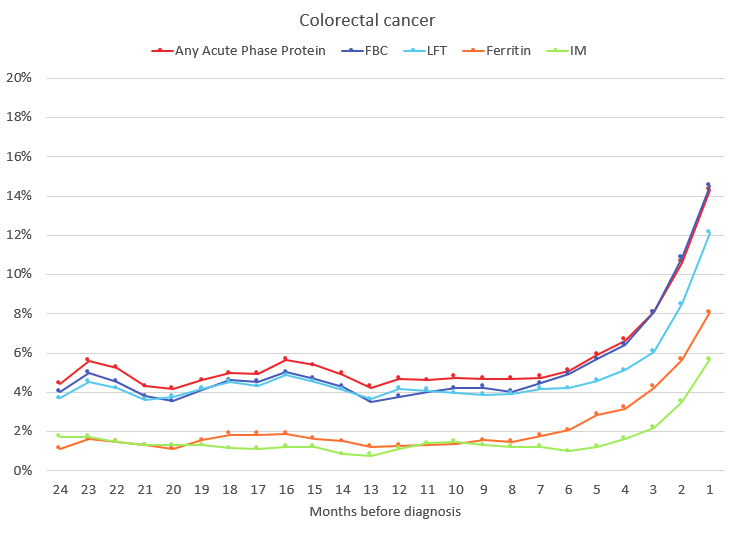

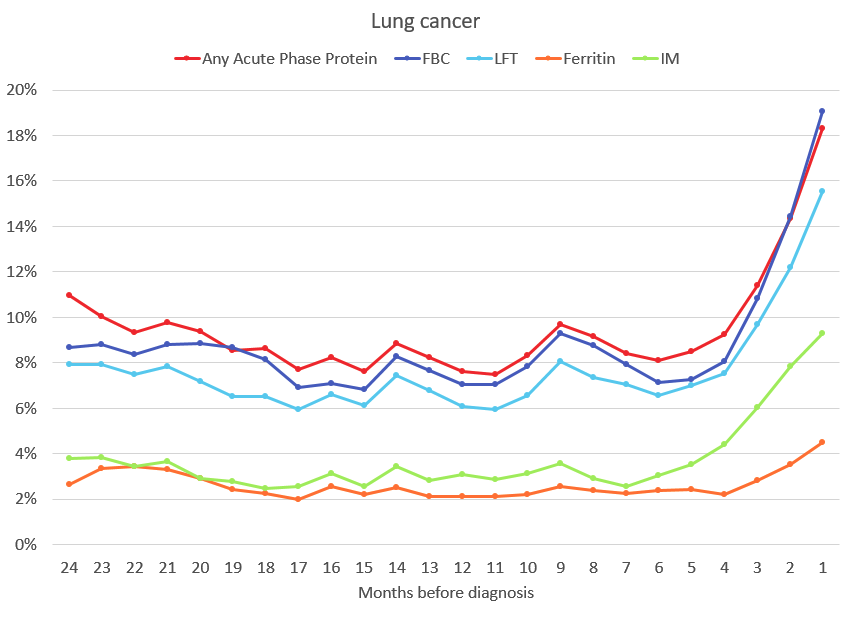


B

A


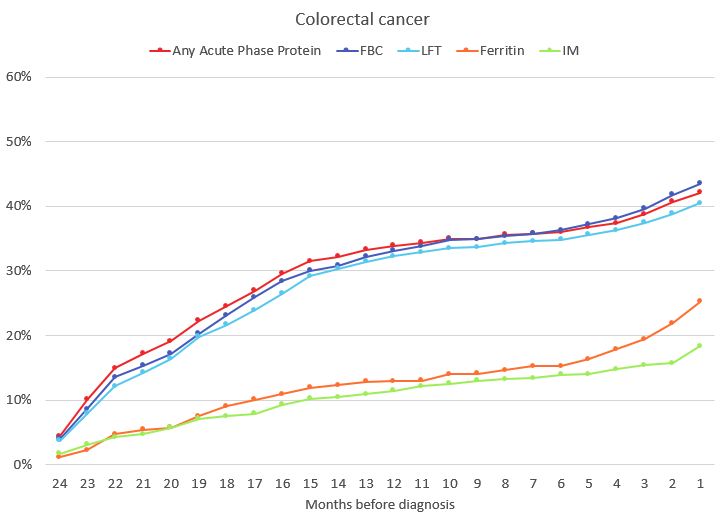

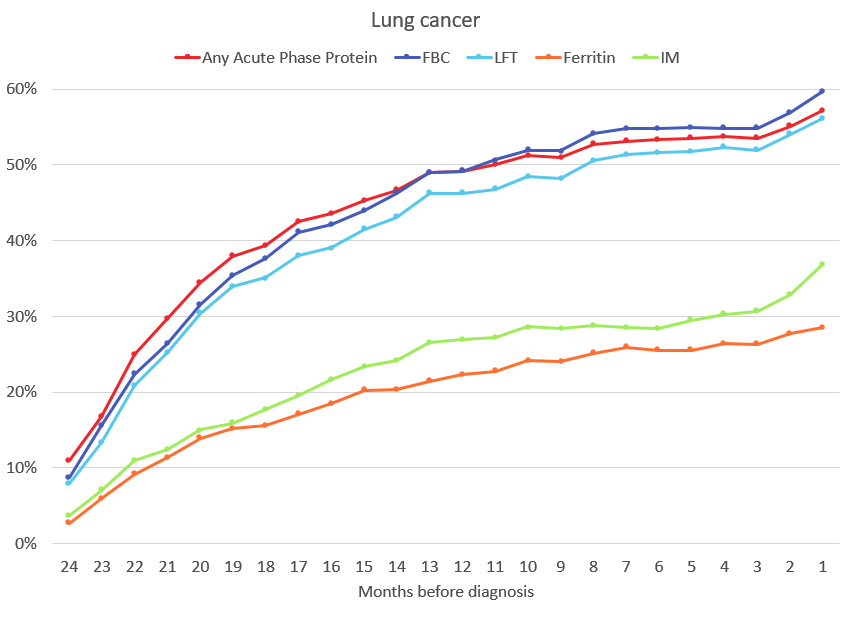


C

D

##### Supplementary Figure S2: Monthly request rates for GP blood tests in colorectal and lung cancer patients in the 24 months before diagnosis (3 month moving average)


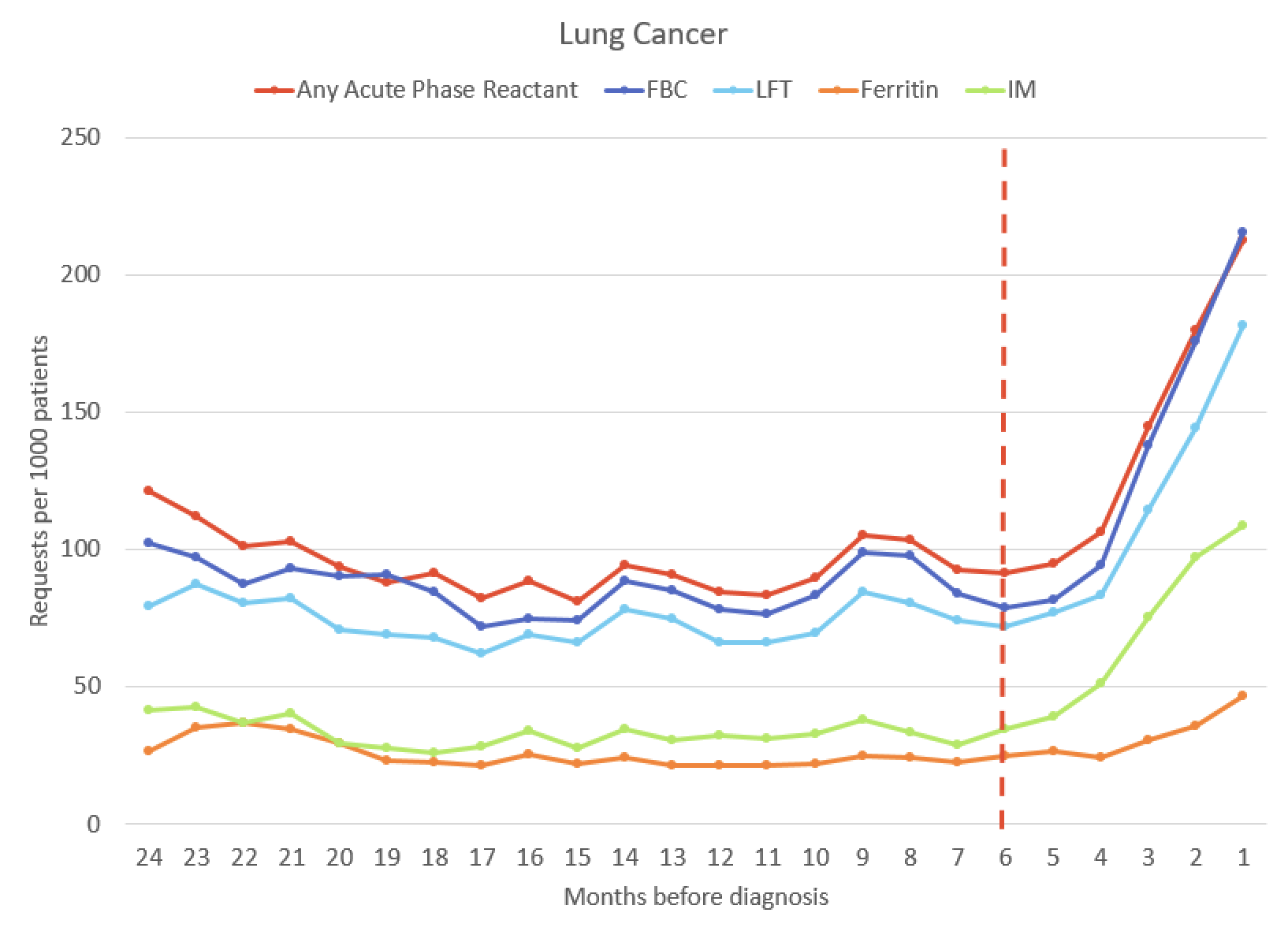

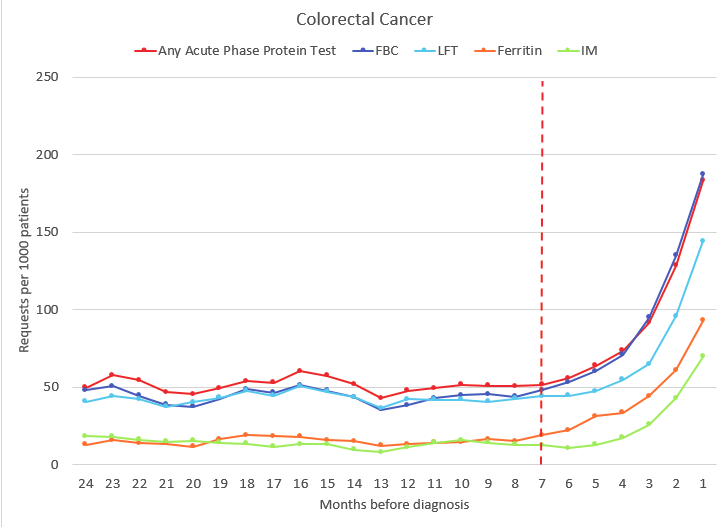
Dashed line represents inflection point for increased test requests identified from modelling; FBC, full blood count; LFT, liver function test; IM, inflammatory marker.

##### Supplementary Table S3: Inflection points for when the rate of blood test requests and abnormalities first start to increase from baseline before cancer diagnosis (identified from statistical modelling)

ESR, erythrocyte sedimentation rate; CRP, c-reactive protein; FBC, full blood count; LFT, liver function test

|  | **Inflection point (months before cancer diagnosis)** | |
| --- | --- | --- |
|  | **Colorectal cancer** | **Lung Cancer** |
| **Blood test requests** | | |
| Ferritin | 7 months | 3 months |
| Inflammatory marker (ESR / CRP) | 5 months | 6 months |
| Any RBCI test (part of the FBC) | 5 months | 4 months |
| Any acute phase reactant test | 5 months | 4 months |
| Albumin (part of the LFT panel) | 4 months | 4 months |
| **Blood test abnormalities** | | |
| Low mean cell haemoglobin concentration | 8 months | 0 months |
| Low mean cell volume | 8 months | 0 months |
| Any abnormal red blood cell indices test | 7 months | 5 months |
| Anaemia | 7 months | 6 months |
| Abnormal ferritin | 7 months | 3 months |
| Low red blood cell count | 7 months | 4 months |
| Any abnormal acute phase reactant test | 7 months | 6 months |
| Raised inflammatory marker | 5 months | 6 months |
| Raised platelet count | 4 months | 5 months |
| Raised total white blood cell count | 4 months | 5 months |
| Raised red blood cell distribution width | 3 months | 6 months |
| Low haematocrit | 3 months | 6 months |
| Low albumin | 3 months | 4 months |

##### Supplementary Table S4: Sensitivity analysis of blood test requests and results excluding screen detected patients

*Percentage out of all patients who had a test result; APR, Acute phase reactant; ESR, erythrocyte sedimentation rate; CRP, c-reactive protein; WBC, white blood cell; RBCI, red blood cell indices; HCT, haematocrit; MCHC, mean cell haemoglobin concentration; MCV, mean cell volume; RBC, red blood cell; RBCDW, red blood cell distribution width.

| **Blood test request in the 12 months before cancer diagnosis** | | |
| --- | --- | --- |
| Blood test | Colorectal cancer patients  (n=855) | Excluding screen detected  (n=729) |
| Any APR test | 327 (38%) | 275 (38%) |
| Platelet | 306 (36%) | 261 (36%) |
| Albumin | 311 (36%) | 259 (36%) |
| Inflammatory marker (ESR/CRP) | 123 (14%) | 115 (16%) |
| Ferritin | 191 (22%) | 161 (22%) |
| Total WBC count | 307 (36%) | 261 (36%) |
| Any RBCI test | 334 (39%) | 281 (39%) |
| Haemoglobin | 334 (39%) | 281 (39%) |
| HCT | 263 (31%) | 225 (31%) |
| MCHC | 232 (27%) | 200 (27%) |
| MCV | 306 (36%) | 260 (36%) |
| RBC count | 297 (35%) | 251 (34%) |
| RBCDW | 213 (25%) | 180 (25%) |
| **Abnormal blood test results in the 12 months before cancer diagnosis** | | |
| Any abnormal APR test* | 177 (54%) | 160 (58%) |
| Raised platelet* | 43 (14%) | 41 (16%) |
| Low albumin* | 22 (7%) | 20 (8%) |
| Raised inflammatory marker (ESR/CRP)* | 84 (68%) | 81 (70%) |
| Raised ferritin* | 22 (12%) | 17 (11%) |
| Low ferritin* | 89 (47%) | 82 (51%) |
| Raised total WBC count* | 43 (14%) | 40 (15%) |
| Any abnormal RBCI test* | 209 (63%) | 182 (65%) |
| Low haemoglobin* | 140 (42%) | 128 (46%) |
| Low HCT* | 110 (42%) | 99 (44%) |
| Low MCHC* | 39 (17%) | 39 (20%) |
| Low MCV* | 58 (19%) | 55 (21%) |
| Low RBC count* | 89 (30%) | 80 (32%) |
| Raised RBCDW* | 140 (66%) | 122 (68%) |

Supplementary Figure S3: Monthly proportion of colorectal and lung cancer patients with an abnormal GP blood test in for the 24 months before diagnosis.

Panels A, B, C and D display incident percentages (3 month moving average) with the dashed line representing the monthly percentage of patients tested, and panels E, F, G and H display cumulative percentages over time. APR, acute phase reactant, RBCI, red blood cell index; IM, inflammatory marker; WBC, white blood cell count; FBC, full blood count, RBCDW, red blood cell distribution width; HCT, haematocrit; RBC, red blood cell; MCV, mean cell volume; MCHC, mean cell haemoglobin concentration

**A**

**B**

*
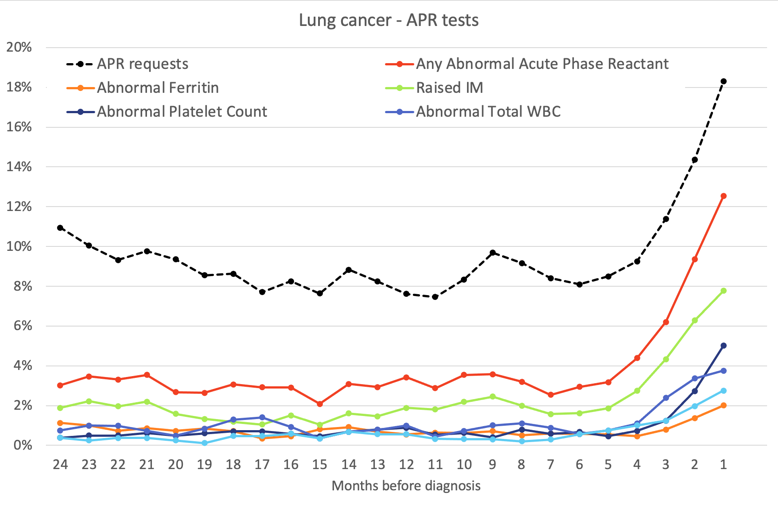

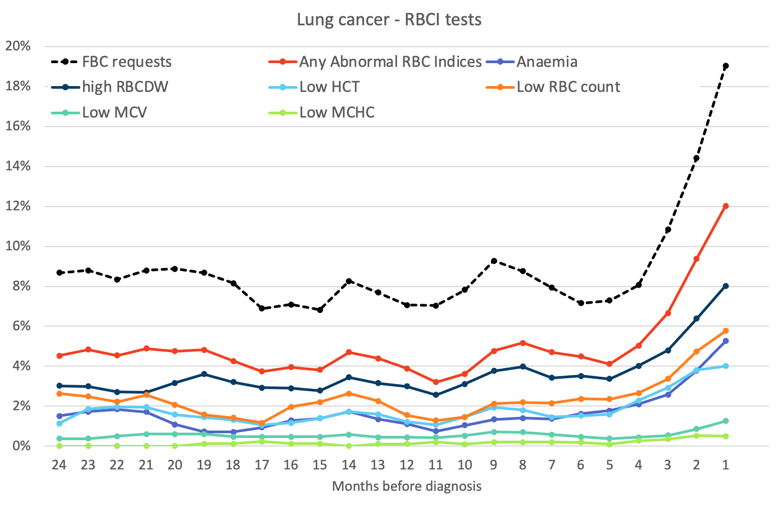
*
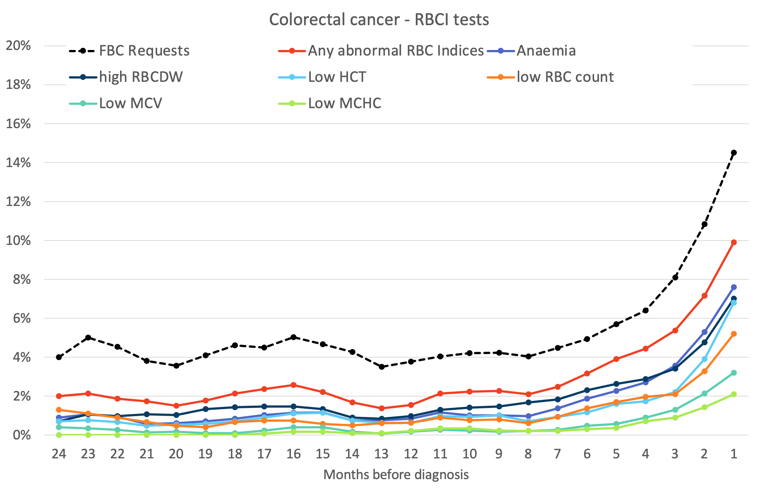

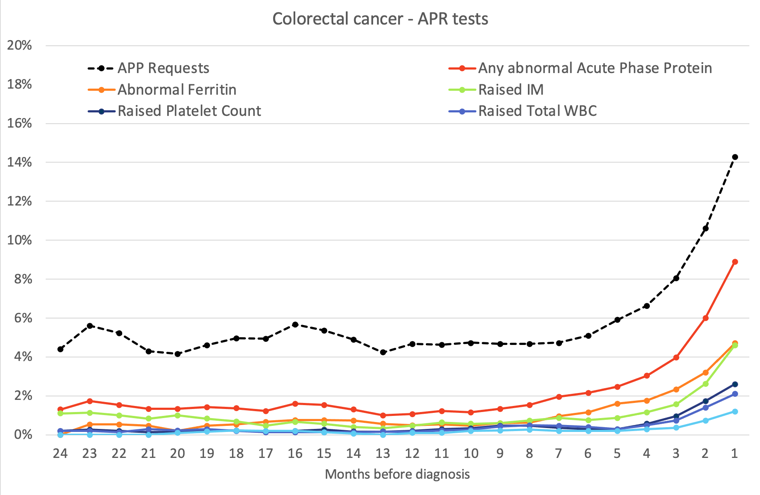


**D**

**C**


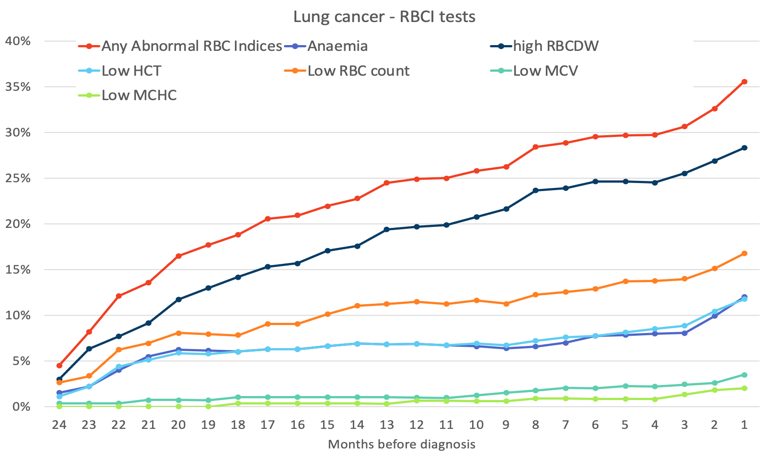

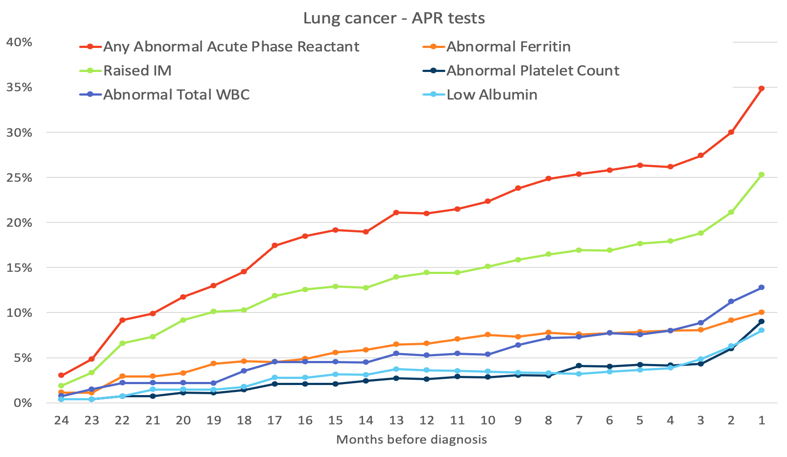

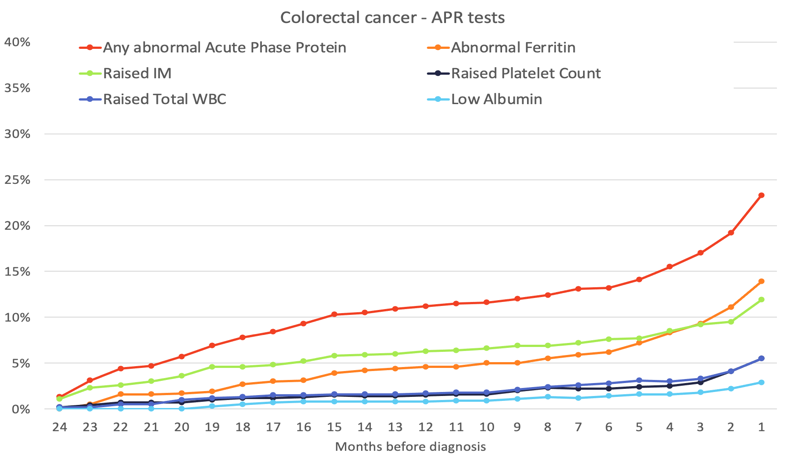

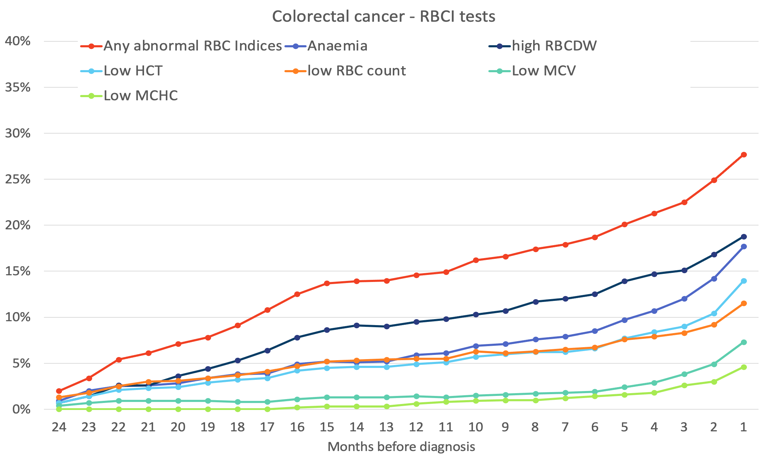


**H**

**G**

**E**

**F**
